# Predicting the outer membrane proteome of *Pasteurella multocida *based on consensus prediction enhanced by results integration and manual confirmation

**DOI:** 10.1186/1471-2105-13-63

**Published:** 2012-04-27

**Authors:** Teerasak E-komon, Richard Burchmore, Pawel Herzyk, Robert Davies

**Affiliations:** 1 Institute of Infection, Immunity and Inflammation, College of Medical, Veterinary and Life Sciences, University of Glasgow, Sir Graeme Davies Building, Glasgow G12 8QQ, UK; 2 Institute of Molecular, Cell and Systems Biology, College of Medical, Veterinary and Life Sciences, University of Glasgow, Joseph Black Building, Glasgow G12 8QQ, UK

## Abstract

**Background:**

Outer membrane proteins (OMPs) of *Pasteurella multocida *have various functions related to virulence and pathogenesis and represent important targets for vaccine development. Various bioinformatic algorithms can predict outer membrane localization and discriminate OMPs by structure or function. The designation of a confident prediction framework by integrating different predictors followed by consensus prediction, results integration and manual confirmation will improve the prediction of the outer membrane proteome.

**Results:**

In the present study, we used 10 different predictors classified into three groups (subcellular localization, transmembrane β-barrel protein and lipoprotein predictors) to identify putative OMPs from two available *P. multocida *genomes: those of avian strain Pm70 and porcine non-toxigenic strain 3480. Predicted proteins in each group were filtered by optimized criteria for consensus prediction: at least two positive predictions for the subcellular localization predictors, three for the transmembrane β-barrel protein predictors and one for the lipoprotein predictors. The consensus predicted proteins were integrated from each group into a single list of proteins. We further incorporated a manual confirmation step including a public database search against PubMed and sequence analyses, e.g. sequence and structural homology, conserved motifs/domains, functional prediction, and protein-protein interactions to enhance the confidence of prediction. As a result, we were able to confidently predict 98 putative OMPs from the avian strain genome and 107 OMPs from the porcine strain genome with 83% overlap between the two genomes.

**Conclusions:**

The bioinformatic framework developed in this study has increased the number of putative OMPs identified in *P. multocida *and allowed these OMPs to be identified with a higher degree of confidence. Our approach can be applied to investigate the outer membrane proteomes of other Gram-negative bacteria.

## Background

The Gram-negative bacterium *Pasteurella multocida *is responsible for economically significant infections of a wide range of animal species. The organism causes a variety of disease syndromes which include pneumonic pasteurellosis of ruminants and pigs, porcine progressive atrophic rhinitis (PAR), fowl cholera, bovine haemorrhagic septicaemia (HS), and human infections via carnivorous bites or scratches [1]. Like all Gram-negative bacteria, the cell envelope of *P. multocida *consists of a symmetrical inner membrane and an asymmetrical outer membrane, separated by the periplasmic space and peptidoglycan layer [2]. The outer membrane consists of an inner phospholipid layer and an outer LPS leaflet [3]. It serves as a selective barrier that controls the passage of nutrients and waste products into and out of the cell and is the interface between pathogen and host. The outer membrane harbours two classes of proteins, integral membrane proteins and peripheral lipoproteins, which together account for 2-3% of the total encoded proteins [4,5]. Integral outer membrane proteins (OMPs) typically have a β-barrel structure whereas lipoproteins are mostly anchored to the inner leaflet of the outer membrane [6,7]. The biosynthesis and translocation of these two groups of proteins have previously been reviewed [6,8-10]. Outer membrane proteins play varied and important roles for bacteria, allowing them to adapt to different environments and host niches [6]. These roles include biogenesis and integrity of the outer membrane, nonspecific porin activity, energy-dependent transport, adherence and membrane-associated enzymatic activity [4]. In *P. multocida*, certain OMPs play important roles in virulence and have been utilized as vaccine antigens [11].

The majority of OMPs can be bioinformatically differentiated and predicted by using their amino acid compositions [12-14], specific protein modifications and sorting mechanisms [15,16], and unique sequences and structural patterns [17-21]. Predictors of outer membrane-located proteins employ a variety of algorithms and methods having different accuracies and sensitivity levels of prediction [22-44]. These predictors can be categorized into three groups: (1) subcellular localization or global predictors which can differentiate between proteins from different compartments; (2) transmembrane β-barrel protein predictors which distinguish β-barrel structures from transmembrane α-helical proteins predominantly found in the inner membrane; and (3) lipoprotein predictors which can discriminate between inner membrane and outer membrane lipoprotein signal peptides [45].

Bioinformatic predictors have been used to identify OMPs in several Gram-negative bacterial species [5,32,46,47]. However, disagreement between the predicted results from individual programs is frequently observed. A combination of different predictors, together with consensus prediction, has been shown to increase the coverage and accuracy of the predicted outer membrane proteome [45,48] including that of transmembrane β-barrel proteins [49]. Heinz *et al. *[50] also employed a manual confirmation step to remove false positives and increase the confidence of the predicted outer membrane proteome.

In a previous *P. multocida *study [51], three predictors (two subcellular localization and one lipoprotein) were used to predict 129 proteins as secreted, outer membrane or lipoprotein from the publicly available genome of *P. multocida *avian strain Pm70 [52]. However, certain predicted proteins were not confirmed as OMPs by all three predictors. Boyce *et al. *[46] identified 35 proteins by proteomics from the *P. multocida *avian strain X-73 but only one third of these proteins were predicted to be OMPs by a combination of two subcellular localization predictors. Therefore, our understanding of the outer membrane proteome of *P. multocida *remains elusive.

In the present study, we used 10 different predictors classified into three groups (subcellular localization, transmembrane β-barrel protein and lipoprotein predictors) to identify putative OMPs from two available *P. multocida *genomes: the avian strain Pm70 and the unannotated genome of porcine non-toxigenic strain 3480. The predicted proteins in each group were filtered by optimized criteria for the consensus prediction and the consensus predicted proteins from each group were integrated into a single list of proteins. We further incorporated a manual confirmation step which included a public database search against PubMed together with various sequence analyses, e.g. sequence and structural homology, conserved motifs/domains, functional prediction, and protein-protein interaction to enhance the confidence of prediction. Using these approaches, we were able to confidently predict the outer membrane proteomes of the two *P. multocida *strains.

## Results

### Prediction of OMPs using different predictors

**Figure 1:**
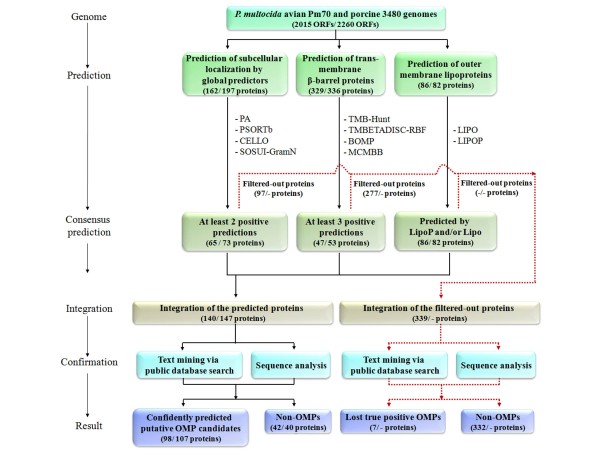
**Overview of the prediction framework**. Diagram representing the workflow of bioinformatic prediction of putative OMPs from the avian and porcine strain genomes of *P. multocida*. Ten predictors were categorized into 3 groups: subcellular localization, transmembrane β-barrel protein prediction and outer membrane lipoprotein prediction. The predicted proteins in each group were filtered by consensus prediction and combined to form a single integrated list. Text mining and sequence analyses were used to confirm that the predicted proteins were outer membrane-associated with a high degree of confidence. The proteins filtered-out from the avian strain genome were also analyzed further by data mining and sequence analysis. The numbers of predicted proteins in each step are shown in parentheses: the first number represents proteins from the avian strain genome and the second number from the porcine strain genome.

Outer membrane proteins were predicted, by ten different bioinformatic programs (Table 1), from the two available genomes of *P. multocida*; the genome of avian strain Pm70 and the genome of porcine strain 3480. These programs were categorized into three groups: subcellular localization predictors (PA, PSORTb, CELLO, SOSUI-GramN), transmembrane β-barrel protein predictors (TMB-Hunt, TMBETADISC-RBF, BOMP, MCMBB), and lipoprotein predictors (LIPO and LIPOP). Individual programs predicted different numbers of proteins. The use of these ten predictors in combination predicted 421 putative OMPs from the avian strain genome (20.9% of the genome) and 439 proteins from the porcine strain genome (19.4% of the genome) (Figure 1, Additional Files 1 and 2).

**Table 1:** Bioinformatic predictors used for the OMP prediction

Predictor group	Programme	Method of predictor
Subcellular localization	Proteome Analyst v. 3.0 (PA)	http://webdocs.cs.ualberta.ca/~bioinfo/PA/[24]
	PSORTb v. 2.0	http://www.psort.org/psortb/[28]
	CELLO v. 2.5	http://cello.life.nctu.edu.tw/[25]
	SOSUI-GramN	http://bp.nuap.nagoya-u.ac.jp/sosui/sosuigramn/sosuigramn_submit.html[39]
Trans-membrane β-barrel structure	TMB-Hunt	http://bmbpcu36.leeds.ac.uk/~andy/betaBarrel/AACompPred/aaTMB_Hunt.cgi[29]
	TMBETADISC-RBF	http://rbf.bioinfo.tw/~sachen/OMPpredict/TMBETADISC-RBF.php[41]
	BOMP	http://services.cbu.uib.no/tools/bomp[31]
	MCMBB	http://athina.biol.uoa.gr/bioinformatics/mcmbb/[23]
Outer membrane lipoprotein	LIPO	http://services.cbu.uib.no/tools/lipo[32]
	LIPOP v. 1.0	http://www.cbs.dtu.dk/services/LipoP/[15]

**Figure 2:**
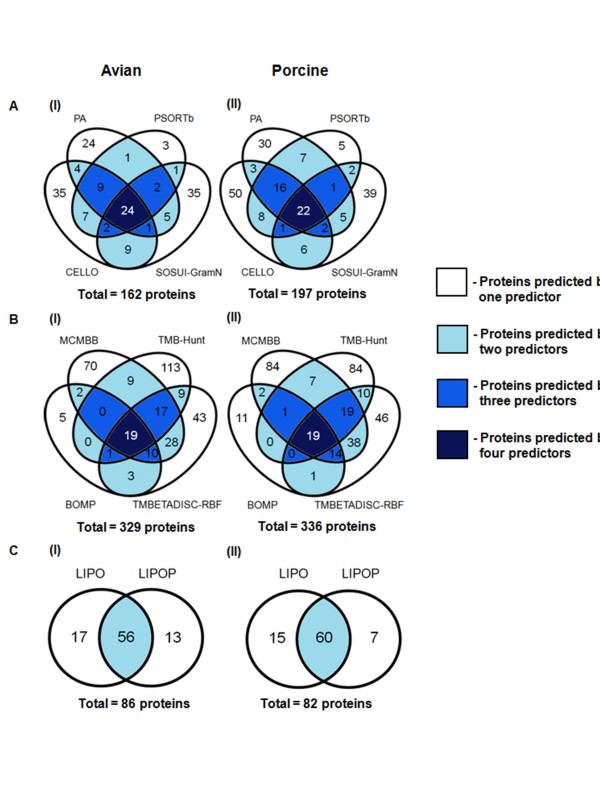
**Within-group comparison of numbers of predicted proteins**. Diagrams show within-group comparisons of the numbers of proteins predicted by three groups of predictors: subcellular localization (A), transmembrane β-barrel protein (B), lipoprotein (C) predictors. The diagrams on the left (i) represent the avian strain genome and those on the right (ii) represent the porcine strain genome. The numbers of proteins predicted by one, two, three or four predictors are indicated.

The subcellular localization predictors identified 162 putative OMPs from the avian strain genome and 197 proteins from the porcine strain genome (Figure 2A). CELLO identified the highest (91 and 108) and PSORTb identified the lowest (49 and 63) number of predicted proteins from the avian and porcine strain genomes, respectively. For the avian strain genome, 97 proteins were predicted by only a single program: 35, 24, 3 and 35 by CELLO, PA, PSORTb and SOSUI-GramN, respectively. Similarly, 124 proteins were identified by a single predictor from the porcine strain genome: 50, 30, 5 and 39 by CELLO, PA, PSORTb and SOSUI-GramN, respectively. Twenty-four proteins were identified from the avian strain genome and 22 from the porcine strain genome using all four programs. The use of two or three programs predicted a total of 41 proteins from the avian strain genome and 51 proteins from the porcine strain genome.

The transmembrane β-barrel protein predictors identified 329 putative β-barrel proteins from the avian strain genome and 336 proteins from the porcine strain genome (Figure 2B). TMB-Hunt identified the highest number of predicted proteins (168) from the avian strain genome, while MCMBB identified the highest number of predicted proteins (184) from the porcine strain genome. BOMP identified the lowest number of predicted proteins (40 and 48) from the avian and porcine strain genomes, respectively. For the avian strain genome, 231 proteins were predicted by only a single program: 70, 113, 43 and 5 proteins by MCMBB, TMB-Hunt, TMBETADISC-RBF and BOMP, respectively. Similarly, 225 proteins were identified by only a single predictor from the porcine strain genome: 84, 84, 46 and 11 proteins by MCMBB, TMB-Hunt, TMBETADISC-RBF and BOMP, respectively. Nineteen proteins were predicted by all four programs in both the avian and porcine strain genomes. The use of two or three programs predicted a total of 79 proteins from the avian strain genome and 92 proteins from the porcine strain genome.

The lipoprotein predictors identified 86 proteins from the avian strain genome and 82 proteins from the porcine strain genome (Figure 2C). LIPO predicted 73 proteins from the avian strain genome and 75 from the porcine strain genome whereas LIPOP predicted 69 proteins from the avian strain genome and 67 from the porcine strain genome. Together, LIPO and LIPOP predicted 56 and 60 proteins from the avian and porcine strain genomes, respectively. However, LIPO identified 17 unique lipoproteins from the avian strain genome and 15 from the porcine strain genome, whereas LIPOP identified 13 unique lipoproteins from the avian strain genome and seven from the porcine strain genome.

**Figure 3:**
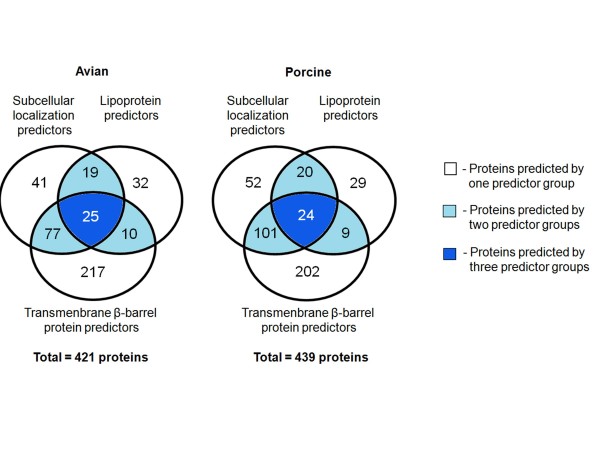
**Between-group comparison of numbers of predicted proteins**. Diagrams show between-group comparisons of the numbers of proteins predicted by three groups of predictors: subcellular localization, transmembrane β-barrel protein, lipoprotein predictors. The diagram on the left side represents the avian strain genome and that on the right the porcine strain genome. Indicated are the numbers of proteins predicted by one, two or three predictor groups.

Comparison of the predicted OMPs by the three groups of predictors revealed that the use of one group of predictors alone identified 290 proteins from the avian strain genome and 283 proteins from the porcine strain genome, whereas a combination of two groups of predictors identified 106 proteins from the avian strain genome and 130 proteins from the porcine strain genome (Figure 3). The use of all three groups of predictors identified 25 proteins from the avian strain genome and 24 proteins from the porcine strain genomes. Noticeably, the transmembrane β-barrel protein predictors predicted a high number of proteins (217 and 202) that were not predicted by the other two groups of predictors.

### Agreement between pairs of predictors

**Figure 4:**
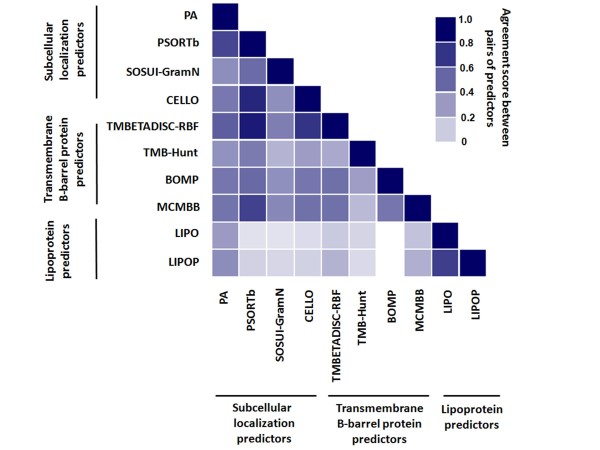
**Analysis of agreement**. Analysis of agreement between pairs of different bioinformatic predictors (the 10 predictors were classified into three groups: subcellular localization, transmembrane β-barrel and lipoprotein predictors) used for the prediction of OMPs within the avian strain genome. Each square represents the color coded agreement score which corresponds to the proportion of commonly predicted proteins for pairs of predictors. The agreement score ranges from 0 for the lowest agreement (white) to 1 for the highest (purple).

The analysis in Figure 4 shows different degrees of agreement between pairs of outer membrane predictors. For the subcellular localization predictors, prediction by pairs of PA and PSORTb as well as PSORTb and CELLO resulted in high agreement scores (0.74 and 0.86, respectively). Pairing of PSORTb with TMBETADISC-RBF and MCMBB also produced high agreement scores (0.90 and 0.76, respectively). For the transmembrane β-barrel protein predictors, predictions by pairing of BOMP with MCMBB and TMBETADISC-RBF as well as MCMBB with TMBETADISC-RBF showed moderate scores (0.57 on average), while pairs of LIPO and LIPOP had a higher agreement score of 0.77 for lipoprotein prediction. The disagreement between lipoprotein predictors and the others was clearly shown with scores of less than 0.5. Subcellular localization predictors discriminate between proteins belonging to different locations. Although these predictors predict a wide range of outer membrane-located proteins, and some of these predictors incorporate the prediction of transmembrane β-barrel proteins and lipoproteins as parts of their programs, some OMPs were possibly mispredicted or excluded, as confirmed by the low agreement score between subcellular localization and lipoprotein predictors. Conversely, the transmembrane β-barrel and lipoprotein predictors differentiate between specific groups of OMPs; they are unable to predict all outer membrane-localized proteins. Therefore, a combination of the subcellular localization, transmembrane β-barrel and lipoprotein predictors resulted in better coverage of the predicted OMPs than the use of a single predictor or group of predictors.

### Consensus prediction

**Figure 5:**
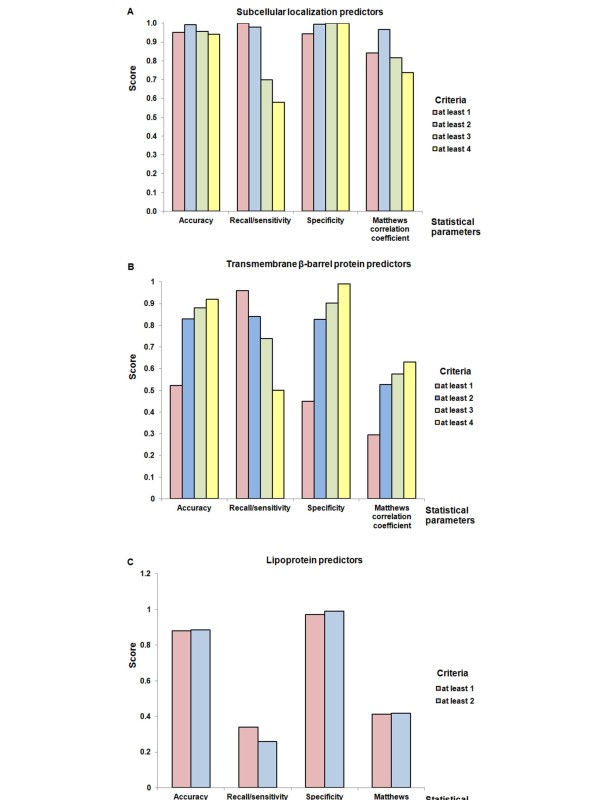
**Criteria for optimization of consensus prediction**. Comparisons of different consensus prediction criteria for the subcellular localization (A), transmembrane β-barrel (B) and lipoprotein (C) predictors tested on a modified PA training dataset using four statistical parameters: accuracy, recall/sensitivity, specificity and Mathews Correlation Coefficient (MCC). Four criteria for subcellular localization predictors, four for transmembrane β-barrel predictors, and two for lipoprotein predictors were considered. The statistical parameters evaluated on each criterion are shown on the x-axis. A score of one (y-axis) indicates the highest performance and zero the lowest.

The above analyses indicated different levels of agreement between pairs of predictors. The use of multiple predictors for subcellular localization, transmembrane β-barrel and lipoprotein predictions produced a large number of predicted proteins many of which were potential false positives. Therefore, the predicted results from individual predictors in each group were filtered using various criteria. The criteria for consensus prediction were first determined using a training dataset containing 526 Gram-negative bacterial protein sequences of known localization (Additional File 3). Measurements of accuracy, recall/sensitivity, specificity and Mathews Correlation Coefficient (MCC) were used to compare the predictive power of the various criteria for consensus prediction [41] and the results are summarized in Figure 5. For the subcellular localization predictors (Figure 5A), all criteria generally presented high accuracy and specificity scores (close to unity). Recall/sensitivity (a measure of ability to identify true positives) decreased as the number of predictors increased, but was close to unity in predictions by at least one or two predictors. The MCC score was highest in the prediction by at least two predictors, whereas those of the other criteria ranged from 0.7 to 0.82. Therefore, prediction by at least two predictors was selected for the subcellular localization predictors because this threshold gave the highest accuracy and MCC scores and very high scores for recall/sensitivity and specificity. For the transmembrane β-barrel protein predictors (Figure 5B), accuracy and specificity scores ranged from 0.8 to 1.0 for predictions by at least two, three or four predictors whereas recall/sensitivity decreased as the criteria threshold increased. Prediction by at least three predictors was chosen for the transmembrane β-barrel protein predictors because this threshold gave the second highest accuracy, specificity and MCC scores with a reasonably high recall/sensitivity score. Although prediction by at least four predictors gave the highest accuracy, specificity and MCC scores, this criterion gave the lowest recall/sensitivity score, indicating potential loss of true-OMPs. For both predictor groups, increased specificity occurred as the number of predictors increased whereas, conversely, recall/sensitivity decreased as the number of predictors increased. For the lipoprotein predictors (Figure 5C), prediction by at least one predictor was selected as this resulted in the highest recall/sensitivity and comparable accuracy, specificity and MCC scores compared with at least two predictors. The selected consensus criteria were next validated on a test dataset (Additional File 4) containing 529 Gram-negative bacterial protein sequences. The results are presented in Table 2. The selected criterion for the subcellular localization predictors yielded very high accuracy, recall/sensitivity, specificity and MCC scores (0.98 to 1.00). The selected criteria for the transmembrane β-barrel protein and the outer membrane lipoprotein predictors showed accuracy and specificity scores higher than 0.9, whereas recall/sensitivity and MCC scores ranged from 0.44 to 0.63. Combining these three selected criteria for the three predictor groups resulted in high accuracy, recall/sensitivity and specificity scores of greater than 0.9. In particular, a recall/sensitivity score of 1.0 indicated that a combination of these three predictor groups identified all of the OMPs from the test dataset.

**Table 2:** Comparison of statistical parameters used for selection of the consensus criteria obtained with the training dataset of 526 Gram-negative bacterial protein sequences of known localization and for validation of the consensus criteria obtained with the test dataset of 529 Gram-negative bacterial protein sequences of known localization

Selected criteria	Statistical parameters			
	
	Accuracy	Recall/sensitivity	Specificity	MCC
**Training dataset**				
**Subcellular localization predictor group**				
At least one positive prediction	0.95	1.00	0.95	0.77
At least two positive predictions	0.99	0.98	0.99	0.94
At least three positive predictions	0.98	0.72	1.00	0.82
All four positive predictions	0.96	0.51	1.00	0.70
**Transmembrane β-barrel protein predictor group**				
At least one positive prediction	0.50	0.95	0.46	0.23
At least two positive predictions	0.86	0.77	0.87	0.46
At least three positive predictions	0.91	0.56	0.95	0.47
All four positive predictions	0.94	0.37	0.99	0.52
**Outer membrane lipoprotein predictor group**				
At least one positive prediction	0.93	0.35	0.98	0.41
All two positive predictions	0.94	0.33	1.00	0.51
**Test dataset**				
At least two positive predictions for subcellular localization predictors	1.00	0.98	1.00	0.98
At least three positive predictions for transmembrane β-barrel protein predictors	0.91	0.63	0.94	0.50
At least one positive prediction for outer membrane protein predictors	0.93	0.44	0.98	0.49
Integration of the three predictor groups	0.92	1.00	0.92	0.69

The *P. multocida *proteins predicted by each group of predictors were filtered using these optimized criteria and resulted in 65 consensus predicted proteins from the avian strain genome and 73 proteins from the porcine strain genome for the subcellular localization predictors; 47 and 53 proteins from the avian and porcine strain genomes, respectively, for the β-barrel transmembrane protein predictors; and 86 and 82 proteins from the avian and porcine strain genomes, respectively, for the lipoprotein predictors (Figure 1). Integration of the consensus-predicted proteins from these three groups subsequently yielded 140 proteins from the avian strain genome and 147 proteins from the porcine strain genome (Figure 1). Of the proteins predicted from the avian and porcine strain genomes, 27 proteins from the avian strain genome and 24 proteins from the porcine strain genome were filtered out by the subcellular localization predictor group but not by the β-barrel transmembrane protein and/or the lipoprotein predictor groups. Similarly, 36 proteins from the avian strain genome and 34 proteins from the porcine strain genome were filtered out by the β-barrel transmembrane protein predictor group but not by the subcellular localization and/or the lipoprotein predictor groups. No proteins from either genome were removed from the lipoprotein predictor group.

### Manual confirmation of the predicted proteins

In the final step, published information available on the predicted proteins was searched, using text mining and sequence analysis, to confirm their outer membrane location. Forty-two proteins (30%) from the avian strain genome and 40 proteins (27%) from the porcine strain genome were removed at the manual confirmation stage. Thirty-one of these proteins were identified in both genomes and included 19 cytoplasmic or inner membrane proteins, 11 periplasmic proteins, two secreted proteins and one phage protein. In this way, 98 proteins from the avian strain genome and 107 proteins from the porcine strain genome were confirmed as being confidently-predicted OMPs (Figure 1). These proteins accounted for 4.9% of the avian strain genome and 4.7% of the porcine strain genome. Details of the confidently predicted OMPs from the avian strain genome are given in Additional File 5. Eighty-nine (91%) of the predicted OMPs in the avian strain genome were also detected in the porcine strain genome, indicating that these two outer membrane proteomes are very similar. Eighteen (17%) of the predicted OMPs from the porcine strain genome had no homologous proteins in the avian strain genome; most of these were hypothetical proteins. However, these proteins included an Omp100 adhesin/invasin homologue in *Aggregatibacter aphrophilus *and two uncharacterized TonB-dependent receptors.

**Figure 6:**
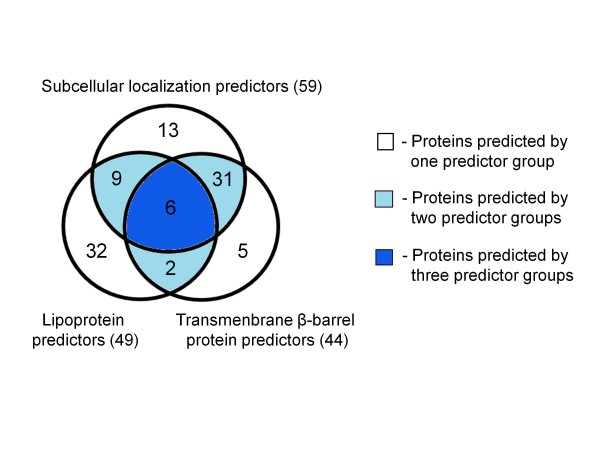
**Comparison of confidently predicted OMPs**. Comparative bioinformatic prediction of the 98 confidently predicted OMPs from the avian strain genome using three different groups of predictors (subcellular localization, transmembrane β-barrel protein and lipoprotein predictors). The predicted proteins in each group were determined as shown in Figure 1. The numbers represent shared or unique predicted proteins. The total number of proteins predicted by each of the three approaches is shown in parentheses.

Of the 98 confidently predicted OMPs of the avian strain genome, 59 (60%) were predicted by subcellular localization, 44 (45%) by transmembrane β-barrel, and 49 (50%) by lipoprotein predictors (Figure 6). Thirty-one proteins were identified as transmembrane β-barrel proteins by both subcellular localization and transmembrane β-barrel predictors. A further five were identified as transmembrane β-barrel proteins by the β-barrel predictors alone; two of these were hypothetical β-barrel proteins (PM0519 and PM1772) which might have novel functions. Thirty-two proteins were uniquely predicted to be outer membrane lipoproteins which were consistent with the agreement analysis. However, almost half of these were of unknown function. A further nine proteins were identified as lipoproteins by both lipoprotein and subcellular localization predictors. Thirteen proteins were identified only by the subcellular localization predictors. Four of these (OmpW and the TonB-dependent receptors PM0745, PM1081 and PM1428) contain transmembrane β-barrel domains but were filtered out by the transmembrane β-barrel predictors since they did not pass the criteria. Two proteins were identified by both transmembrane β-barrel and lipoprotein predictors and six proteins by all three groups of predictors.

**Figure 7:**
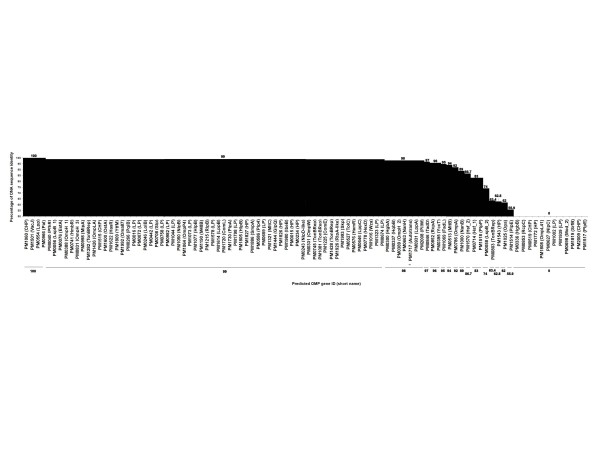
**Comparison of DNA sequence identity of the confidently predicted OMPs from the avian and porcine strain genomes**. DNA sequences of the confidently predicted OMPs from the avian strain genome were compared to the confidently predicted proteins from the porcine strain genome using BLAST. The percentage of identity (y-axis) was plotted against the *P. multocida *avian strain gene IDs and short protein names in parentheses (x-axis). CHP, TonBRep, HP, LP and Autotrans are abbreviations for conserved hypothetical protein, TonB-dependent receptor, hypothetical protein, lipoprotein and autotransporter, respectively. Numbers above the graph indicate the percentage of identity and OMPs are grouped according to the same percentage of identity.

Comparison of the DNA sequence identity of the confidently-predicted OMPs from the avian and porcine strain genomes indicated that the majority (64 proteins) of the predicted OMPs had sequence identities above 99% (Figure 7). OMPs having DNA sequence identities less than 99% included HgbA (98%) and HgbB (haemoglobin and haemoglobin-haptoglobin receptors, 98%), OmpH_2 (a porin, 98%), NanH (sialidase, 98%), PM1717 (an autotransporter, 98%), LppA (98%), PilW (98%), TadD (97%), RcpA (96%), YccT (96%), FadL (95%), MltB (94%), OmpA (92%), NanB (89%), Hsf_2 (trimeric autotransporter, 87%), Hsf_1 (83%), PlpP (83%), LspB_2 (an autotransporter, 74%), PM0803 (TonB-dependent receptor, 63%), PM1543 (hypothetical protein, 63%), Opa (62%) and PlpE (56%).

### Analysis of filtered-out predicted proteins

Proteins of the avian strain genome that were filtered out at the consensus prediction stage were integrated and further analyzed by manual confirmation (Figure 1). Ninety-seven (60%) of the proteins predicted by the subcellular localization predictors and 277 (84%) of the proteins predicted by the transmembrane β-barrel predictors were filtered out; no proteins were filtered out by the lipoprotein predictors. In total, 339 of the proteins predicted by the subcellular localization and transmembrane β-barrel protein predictors were filtered out by consensus prediction. Further analyses of these proteins by manual confirmation revealed that 39 were putative OMPs and/or periplasmic proteins. However, 20 of these had previously been predicted by the lipoprotein predictor group and were therefore removed from the list of filtered-out proteins. A further six proteins that had previously been filtered out by the transmembrane β-barrel predictor group had passed the criteria of the subcellular localization predictor group and were also removed from the list of filtered-out proteins. Thus, 13 (4%) of the filtered-out proteins likely represented true OMPs. Manual confirmation of these 13 proteins showed that seven were indeed putative OMPs. These included HbpA/DppA (PM0592), NlpD (PM1507), RcpB (PM0851), MltA (PM0928), ComEA (PM1665), NlpI (PM1113) and a putative OMP (PM1623). The remaining six proteins were putative periplasmic proteins. Thus, only seven (2%) of the 339 filtered-out proteins were putative OMPs, whereas 332 (98%) proteins could be confidently classified as non-OMPs. These results clearly demonstrate that consensus prediction removes the majority (98%) of non-OMPs and substantially reduces the time that needs to be spent on manual confirmation.

### Functions of the confidently predicted OMPs

The functions of the 98 confidently predicted OMPs in the avian strain genome are shown in Additional File 6. These functions include outer membrane biogenesis and integrity (12 proteins), transport and receptor (25 proteins), adherence (7 proteins) and enzymatic activity (9 proteins). Forty-one proteins have unknown functions (although 17 are named) and 27 of these are lipoproteins. Interestingly, two or three copies of genes encoding certain proteins were predicted. For example, three *ompH *genes and two genes of *lspB, hsf, hgbB, plpE *and *hlpB *were predicted. Similar observations, including three *ompH *genes and two genes of *lspB, hsf, hgbA*, and *plpE*, were made for the porcine strain genome. Two proteins, HexD and Wza, were predicted from both genomes but they appear to have similar functions in capsular polysaccharide transport. Twelve TonB-dependent receptors including HemR (hemin receptor), PfhR and HasR (heme receptors), HmbR, HgbA and two HgbB (haemoglobin receptors), and PM0803, PM1075, PM1081, PM1282 and PM1428 were predicted in the avian strain genome; notably, most of these are involved in iron uptake. Similarly, 14 TonB-dependent receptors were identified in the porcine strain genome including HemR, PfhR and HasR, HmbR, two HgbAs, HgbB, PM0803, PM1075, PM1081, PM1282, PM1428 and two uncharacterized porcine strain-specific proteins (PMpPor1882 and PMpPor2194).

### Physicochemical properties of putative OMPs

Analysis of physicochemical parameters (Additional File 5) highlighted the properties of the putative OMPs. The predicted proteins had molecular masses ranging from 7.1 to 276.2 kDa (52.4 ± 43 kDa average) and an average pI value of 8.1 ± 1.5. The average size of the predicted lipoproteins was smaller than that of the other proteins. Some proteins had very large sizes such as Hsf_1 (276 kDa) and the hypothetical lipoprotein PM0659 (214 kDa). The average GRAVY score [53] was -0.35 ± 0.24 which indicated that the proteins were relatively hydrophilic compared to the predicted inner membrane proteins (data not shown). The predicted OMPs had more β-sheet strands (3-44 strands) than α-helices (0-3 helices).

## Discussion

### Different prediction methods

Each prediction method used in the present study (Table 1) is based on different algorithms and training datasets. The subcellular localization predictors aimed to determine all cellular components (secreted, outer membrane, periplasmic, inner membrane and cytoplasmic proteins) of the genome of *P. multocida*. PA analyzes keywords obtained from various databases using machine-learned classifiers and provides a user-friendly graphical explanation of each prediction [24]. PSORTb combines multiple prediction components and each component performs a specific function including homology prediction, transmembrane prediction, a signal peptide prediction, and a specific motif prediction [28]. SOSUI-GramN uses only the total sequence and physicochemical properties of the N- and C-terminal signal sequences for its prediction [39]. CELLO uses a supervised-learning method (support vector machines, SVMs) to detect specific amino acid compositions and motifs [25]. Of 162 proteins predicted by the subcellular localization predictors from the avian strain genome, 15% were predicted by all four predictors, 25% by two or three predictors and 60% by a single predictor. Similarly, of 197 predicted proteins from the porcine strain genome, 11% of proteins were predicted by all four predictors, 26% by two or three predictors and 63% were predicted by a single predictor. Therefore, approximately 40% of the proteins predicted by the subcellular localization predictors were predicted by at least two predictors. Although PA and PSORTb have been widely used and reported as highly efficient predictors [54], SOSUI-GramN and CELLO identified additional OMPs (e.g., RcpC, NanB, TadD, LppC and PM1515) which the first two predictors did not. Overall, the use of multiple subcellular localization predictors increased both the prediction coverage and the confidence of prediction.

Conversely, the transmembrane β-barrel protein and lipoprotein predictors identified specific groups of OMPs. The four transmembrane β-barrel protein predictors discriminate between β-barrel proteins and non-β-barrel proteins. BOMP detects the C-terminal signal sequence and typical β-barrel pattern of the total amino acid sequence [31]. MCMBB uses a fast algorithm to determine alternating patterns of the transmembrane β-barrel proteins [23]. TMB-Hunt and TMBETADISC-RBF identify transmembrane β-barrel proteins based on amino acid composition profiles using different algorithms [29,41]. MCMBB and TMB-Hunt predicted more proteins than BOMP and TMBETADISC-RBF (Figure 2, Additional Files 1 and 2). The explanation for this could be due to differences in the algorithms, scoring schemes and performance levels. By using these four transmembrane β-barrel protein predictors, 30% and 33% of transmembrane proteins were predicted by at least two predictors from the avian and porcine strain genomes, respectively; the remaining transmembrane proteins were predicted by a single predictor. The use of multiple transmembrane β-barrel protein predictors again resulted in an increase in the confidence of prediction.

For the lipoprotein predictors, LIPO and LIPOP detect outer membrane lipoproteins by using their conserved lipo-box sequences. Together, LIPO and LIPOP predicted 65% of lipoproteins from the avian strain genome and 73% of lipoproteins from the porcine strain genome. These results indicate a high level of agreement between the two predictors and a high level of confidence.

Our findings confirm results obtained with *Escherichia coli *which showed that the use of multiple predictors increases the efficiency of subcellular localization prediction as well as specific-feature (β-barrel and lipid modified proteins) prediction when compared with the use of a single program [45]. Mirus and Schleiff [17] compared different transmembrane β-barrel protein predictors and showed that the combinatory approach improved the reliability of the prediction. Moreover, we have also confirmed that the combined use of different predictors improves the coverage of predicted OMPs and our findings are consistent with previous work in other bacterial species [5,32,47]. However, a higher number of predictors were used in the present study.

### Filtration, integration and confirmation of the prediction results

In the present study, we used a combination of subcellular localization, transmembrane β-barrel protein and lipoprotein predictors, followed by consensus prediction with optimized criteria, integration and manual confirmation (data mining and sequence analyses) to predict OMPs in the available avian and porcine *P. multocida *genomes. Consensus prediction was validated on a modified PA dataset containing 1055 Gram-negative bacterial protein sequences (of < 25% similarity) which were divided into training and test datasets. The consensus criteria were selected by comparing statistical parameters obtained using various criteria in the training dataset; the selected criteria were validated using the test dataset. The selected criteria were chosen to optimize or maximize the accuracy, recall/sensitivity, specificity and MCC scores. The criterion (i.e. at least two predictors) selected for the subcellular localization predictors was chosen by maximizing the accuracy, specificity and MCC scores (Figure 5 and Table 2). For the transmembrane β-barrel protein predictor group, the accuracy, specificity and MCC scores increased as the number of predictors increased (i.e. maximum values were achieved for at least 4 predictors). However, there was a corresponding reduction in the recall/sensitivity scores (Figure 5). Thus, although more false-positive proteins were removed as the number of predictors increases, there was also an increase in the loss of true positive proteins. Therefore, the consensus criterion (at least three predictors) for the transmembrane β-barrel protein predictors was selected by optimization of recall/sensitivity in conjunction with the other statistical parameters. The MCC scores of the selected criteria for the transmembrane β-barrel protein and the outer membrane lipoprotein predictors were moderate (Figure 5). The reason for these moderate scores could be due to the ability to predict specific subgroups of OMPs (e.g. transmembrane β-barrel and outer membrane lipoprotein) with these predictors. When the three groups of predictors were combined, the prediction performance was enhanced (Table 2).

Applying the consensus prediction to the predicted OMPs of *P. multocida *significantly reduced the number of false positive proteins, but this advantage has to be measured against the loss of a small number (seven) of true positive proteins. If absolutely necessary, the identities and locations of the filtered-out proteins can be checked using the additional manual confirmation step (Figure 1). Applying the consensus method and manual confirmation enhances the confidence and reliability of the predicted proteins [45,48,50]. Viratyosin *et al. *[48] developed a computational framework incorporating consensus prediction of the subcellular localization predictors and homology information for subcellular localization prediction of the *Leptospira interrogans *genome and identified 63 putative OMPs. Similarly, Heinz *et al. *[50] used multiple prediction phases, including screening of the inner membrane proteins, manual confirmation of the PSORTb database, and prediction of β-barrel, β-helix and lipoproteins, to identify the OMPs in *Chlamydiae*. Our study provides a simple framework which improves the confidence of prediction of the outer membrane proteome of *P. multocida *compared to previous studies.

By using the consensus prediction followed by integration of the results for three predictor groups (Figure 1), the number of predicted proteins decreased from approximately 400 to 140 for the avian strain genome and to 147 for the porcine strain genome. The manual confirmation step further reduced the numbers to 98 and 107 confidently-predicted putative OMPs for the avian and porcine *P. multocida *genomes, respectively. These values represent an average of 4.8% of the total proteome. The two predicted outer membrane proteomes were very similar, sharing 89 (83%) proteins. The majority (64) of these proteins had sequence identities above 99%, whereas 22 proteins had sequence identities in the range of 55.9 to 98%. Twelve proteins were present in either the avian or porcine genomes but not both. Of these, only three, namely, the Omp100 adhesin/invasin and two uncharacterized TonB-dependent receptors, were annotated as having putative function, in adherence and transport. The presence of these proteins in porcine isolates alone suggests a possible role in host adaptation.

We compared the confidently predicted putative OMPs from the avian strain genome obtained by our prediction framework with those predicted by a recently published subcellular localization predictor ClubSub-P which was developed based on cluster-based and consensus prediction methods [55]. Fifty-eight proteins were predicted by the ClubSub-P program including 34 inner or outer membrane lipoproteins, 20 transmembrane β-barrel proteins and four lipidified transmembrane β-barrel proteins. Forty-eight of these proteins were predicted by our prediction framework, whereas ten proteins were not predicted by our method. The ClubSub-P predictor did not discriminate between outer and inner membrane lipoproteins. The ten proteins not predicted by our method were removed during either the consensus prediction or manual confirmation step. Therefore, our prediction framework provided better coverage of the predicted outer membrane proteome of *P. multocida *compared to ClubSub-P [55].

Of the 98 confidently predicted putative OMPs from the avian strain genome, 48 proteins were predicted by at least two groups of predictors, while the remainder were identified by only one approach (Figure 6). We were able to classify the predicted OMPs into transmembrane β-barrel, lipidified transmembrane β-barrel, and lipidified proteins. The subcellular localization predictors predicted four potential β-barrel proteins that were filtered out by the β-barrel predictor group. The loss of these potential true OMPs in the prediction may have occurred due to the stringent criteria used during the consensus prediction as increased stringency reduces the rate of false positives at the cost of an increased rate of false negatives. The manual confirmation of individual predicted proteins helped in the elimination of the false-positive proteins, such as some secreted and periplasmic proteins, and confidently confirmed that predicted proteins were targeted to the outer membrane. Moreover, it also assigned relevant functions to about 60% of the predicted proteins whose roles included outer membrane biogenesis and integrity, transport and receptor functions, adherence, and enzymatic activity. However, the remainder of the proteins, especially the lipoproteins, are hypothetical and require further characterization.

**Figure 8:**
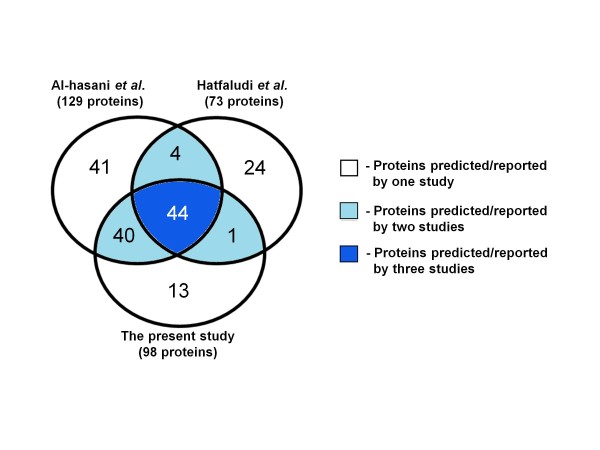
**Comparison of OMPs predicted in the present study to those in previously published research**. Diagram comparing the numbers of OMPs predicted in the present study with those predicted by Al-hasani *et al. *[51] and reported by Hatfaludi *et al. *[56]. Indicated are the numbers of proteins predicted/reported by one, two or all three studies. The total number of proteins predicted/reported by each of the three studies is shown in parentheses.

Eighty-four of the 98 putative OMPs predicted from the avian strain genome in the present study were also identified in the previous study by Al-hasani *et al. *[51] (Figure 8). These authors predicted 129 putative OMPs and secreted proteins from the avian *P. multocida *genome using only three predictors (PA, PSORTb and LIPOP). The additional 14 proteins that we identified included seven proteins predicted by transmembrane β-barrel protein predictors (a pilus assembly protein RcpC, a sialidase NanB, Mce/PqiB, YccT, PM0519, PM1515, PM1772), three proteins predicted by lipoprotein predictors (PM1002, PM1798, PM1939), three proteins predicted by subcellular localization predictors (PM0015, PM0234, a RlpA-like protein PM1926), and one protein (PM1323) predicted by all these predictor groups. In contrast to the present study, Al-hasani *et al. *[51] did not apply consensus prediction to filter their predicted results and were interested in identifying both OMPs and secreted proteins. Consequently, there was disagreement in the localization of 19 proteins between the three predictors (particularly between PA and PSORTb) and these proteins could not be concluded to be OMPs with certainty. Forty-five proteins predicted by Al-hasani *et al. *[51] were not confidently predicted in the present study (Figure 8). Of these, 18 were not predicted and 27 were filtered out by consensus prediction or manual confirmation. Clearly, the use of a large number of predictors, together with consensus prediction, allowed us to identify a larger number of outer membrane-associated proteins with a greater degree of confidence.

Hatfaludi *et al. *[56] reviewed the functions and classification of the OMPs of *P. multocida *and reported that 73 proteins were outer membrane-located based on previously published experimental research. We have confidently predicted 45 of these proteins, whereas 28 proteins were not predicted in the present study (Figure 8). One protein, TbpA, was not identified because of its absence from the avian and porcine strain genomes. The remaining 27 proteins were not included in our list of confidently predicted OMPs for a number of reasons. Nine proteins were filtered out by consensus prediction (five proteins) or shown to be non-OMPs by manual confirmation (four proteins). The proteins that were filtered out by consensus prediction included lipoprotein PM0979, a competence-related DNA-binding and uptake protein ComE1, an outer membrane-bounded sialic acid-binding protein NanP or YiaO, and Flp (Tad) operon proteins Flp1 and RcpB. The remaining 18 proteins were not identified as OMPs by any of the ten predictors in the present study. These included cytoplasmic proteins (3), inner membrane proteins (4), a periplasmic protein (1) and extracellular proteins (2). There are a number of explanations for the presence of these proteins in the list assembled by Hatfaludi *et al. *[56] including contamination during outer membrane extraction and multiple subcellular localizations of certain proteins. Significantly, of the 98 OMPs predicted from the avian strain genome in the present study, 53 OMPs (Figure 8) were not reported by Hatfaludi *et al. *[56]. These included OmpH_3, Opa, Hsf_1 and _2, LolB, LppA, RlpB, PlpE, SmpA, Plp4, LppC, HexD and Wza. Clearly, these findings indicate that there is still a lack of experimental evidence relating to the structures and functions of the majority of the predicted outer membrane proteome.

Both Hatfaludi *et al *[56] and Al-hasani *et al *[51] identified the same 44 proteins that were also predicted in the present study (Figure 8). However, a further 40 proteins in our list were only predicted by Al-hasani *et al *[51] whereas one protein was only reported by Hatfaludi *et al *[56]. In the present study, we have predicted 13 proteins that were not described by Hatfaludi *et al *[56] or predicted by Al-hasani *et al *[51]. These include the Flp operon protein RcpC, the paraquat-inducible protein Mce/PqiB, YccT, a RplA-like protein PM1986, and nine hypothetical proteins (PM1515, PM0519, PM1772, PM1002, PM1798, PM1939, PM0015, PM0234 and PM1323). However, the functions of certain of these proteins have not been determined. Overall, the present study has improved the coverage of the predicted outer membrane proteome of *P. multocida *by 15% compared to that of Al-hasani *et al *[51]. Our simple prediction framework has allowed us to confidently predict and increase the coverage of the outer membrane sub-proteome of *P. multocida *by using currently available predictors and databases. However, our ten selected predictors could be replaced or modified as improved subcellular localization or specific-feature predictors are made available. For example, the Freeman-Wimley prediction algorithm was developed to improve the prediction of transmembrane β-barrel proteins over previous algorithms to an accuracy of 99% and MCC score of 0.75 [57]. Freeman and Wimley [57] demonstrated that their prediction algorithm was more accurate than BOMP and TMBETADISC-RBF, two of the methods used in the present study. This method could potentially be incorporated into our transmembrane β-barrel protein predictor group to further improve the prediction performance of our prediction method if it was available as an online user-friendly tool for genome-scale prediction. However, it should be pointed out that of the seven putative OMPs that were filtered out by consensus prediction and subsequently identified by data-mining, five (HbpA/DppA, NlpD, ComEA, Nlpl and PM1623) were transmembrane β-barrel proteins that were identified by the transmembrane β-barrel protein predictors used - BOMP (3 proteins), MCMBB (2), TMB-Hunt (1) or TMBETADISC-RBF (1). If the Freeman-Wimley method identified all of these, only ComEA and PM1623 would pass the consensus prediction criteria (i.e. prediction by at least three predictors). Recently, Goudenege *et al. *[58] created a subcellular localization database, CoBaltDB, for Bacteria and Archeae by incorporating 43 different predictors and 784 complete proteomes, but they did not give consensus localization of the predicted proteins and a decision for protein location has to be made by the users themselves. By using this database, our prediction framework could also be applied to confidently predict subcellular localization in other bacterial species.

## Conclusions

In the present study, we have designed a simple prediction framework that allows the prediction of putative OMPs from the available *P. multocida *genomes with a high level of confidence. Our approach involves the use of multiple predictors divided into three groups, together with consensus prediction followed by integration and manual confirmation. We have confidently identified 98 putative OMPs from the avian strain genome and 107 putative OMPs from the porcine strain genome of *P. multocida *with 83% overlap between the two genomes. The coverage of the outer membrane proteome of this bacterium has been improved on previous research. The identification of previously unrecognized OMPs in strains of *P. multocida *from different host species will stimulate further studies into the molecular basis of the pathogenesis of this organism. In a separate study, the authors have analyzed the outer membrane proteomes of eight *P. multocida *isolates using complementary proteomic methods [59]. In this study, more than half of the predicted outer membrane proteome has been demonstrated experimentally. Fifty-four putative OMPs have been identified representing 52% of the predicted avian outer membrane proteome and 48% of the predicted porcine sub-proteomes. This study not only provides a basis for furthering our understanding of the outer membrane proteome of *P. multocida *but can also be applied to other Gram-negative bacteria.

## Methods

### *P. multocida *genomes

The publicly available genome of the avian *P. multocida *subsp. *multocida *strain Pm70 [GenBank: AE004439] and the unannotated genome of the porcine non-toxigenic *P. multocida *strain 3480 [Project ID: 32177] were used for all bioinformatic analyses. The avian strain genome containing 2,015 protein-coding genes was retrieved from NCBI. The 2,260 protein-coding genes of the unannotated porcine genome (kindly provided by Dr. A. Gillaspy, University of Oklahoma) were manually predicted using GeneMark [60] and automatically named using Blast2GO [61].

### Selection of bioinformatic predictors

The scheme for the bioinformatic prediction of the OMPs is shown in Figure 1. Three groups of predictors, involving 10 genome-scale programs (Table 1), were used to predict putative OMPs from the two genomes. Subcellular localization or global predictors included the programmes Proteome Analyst (PA) [24], PSORTb [28], CELLO [25] and SOSUI-GramN [39]; transmembrane β-barrel protein predictors included TMB-Hunt [29], TMBETADISC-RBF [41], BOMP [31] and MCMBB [23]; and outer membrane lipoprotein predictors included LIPO [32] and LIPOP [15]. Predicted results of each protein in the HTML or Excel formats from individual programmes were parsed using in-house built perl scripts.

### Analysis of agreement between pairs of bioinformatic predictors

The advantages of using multiple predictors over a single predictor can be evaluated by analysis of agreement between pairs of selected programs [45]. This analysis determines the level of agreement between different predictors by use of the following formula:

$Agreement\;\left(A\right)=\frac{{\left(P_{1}\cap P_{2}\right)}_{L}}{P_{L}^{\prime }}$[45]

where (*P_1 _*∩ *P_2_*)*_L _*is the number of predicted proteins shared between predictor *P_1 _*and *P_2 _*for a subcellular location, *L*, and *P'_L _*is the total number of predicted proteins from a lower coverage program of the predictor pair for that location. An agreement score (*A*) of one means that all proteins predicted by both methods (*P_1 _*and *P_2_*) are localized on a location, *L*. A score of zero means that there are no shared predicted proteins between the two predictors for a location, *L*. In-house built perl scripts were used to analyze the predicted results of each program before pairwise comparison and calculation of the agreement score.

### Criteria optimization, consensus prediction and integration

Predicted proteins from different programs in each group were filtered by consensus prediction (Figure 1) with optimized criteria to eliminate redundancy and proteins of low confidence. These analyses were performed using Excel. We varied the criteria by increasing the number of positive predicted results as a threshold in each predictor group. For the subcellular localization and transmembrane β-barrel predictor groups, the criteria were varied from positive predicted proteins obtained by at least one, two, three or four predictors. For the lipoprotein predictors, the criteria were varied from positive predicted proteins obtained by at least one or two predictors. The effectiveness of these criteria was validated by two-fold cross-validation using two different protein data sets (training and test datasets) modified from the PA protein dataset http://webdocs.cs.ualberta.ca/~bioinfo/PA/. The original PA dataset contained 6089 Gram-negative bacterial protein sequences of known subcellular locations including cytoplasmic (3959), inner membrane (1127), periplasmic (400), outer membrane (361) and extracellular (242) proteins. These protein sequences were clustered using the minimum identity of 25% by the BLASTclust program http://toolkit.genzentrum.lmu.de/blastclust#. The percentages of sequence identity and similarity of pairwise comparison of these sequences were generated by the MatGat program [62]. The sequences which had sequence identity greater than or equal to 25% were excluded. This modified PA dataset containing 1055 protein sequences was then sub-divided randomly into two, a training dataset and a test dataset. The training dataset contained 526 protein sequences of known subcellular locations including cytoplasmic (157), inner membrane (219), periplasmic (71), outer membrane (43) and extracellular (36) proteins. The test dataset contained 529 protein sequences of known subcellular locations including cytoplasmic (158), inner membrane (220), periplasmic (71), outer membrane (43) and extracellular (37) proteins. Pairs of proteins between and within these two datasets had sequence identities of less than 25%. Different consensus criteria of the three predictor groups were trained using the training dataset. We used calculations of accuracy, recall/sensitivity, specificity and Matthews Correlation Coefficient (MMC) from the training dataset to assess the prediction performance using these different criteria. Accuracy of prediction is defined as the degree of closeness of the predicted number of OMPs compared to the actual number of OMPs. Recall or sensitivity is defined as the proportion of OMPs that are correctly predicted. Specificity is defined as the proportion of non-OMPs that are correctly predicted. MCC measures sensitivity and specificity together and ranges from -1 to 1, where MCC = 1 indicates a perfect prediction, MCC = 0 indicates a completely random prediction, and MCC = -1 indicates a reverse correlation. The formulae are shown below where TP represents the number of true positives (proteins predicted as OMPs being OMPs), TN the true negatives (proteins predicted as non-OMPs being non-OMPs), FP the false positives (proteins predicted as OMPs being non-OMPs) and FN the false negatives (proteins predicted as non-OMPs being OMPs). This optimization tended to reduce most of the false positive and retain most of the true positive proteins.

$$\begin{array}{c} Accuracy\;\left(\%\;of\;correct\;predictions\right)=\frac{\left(TP\;+\;TN\right)}{\left(TP\;+\;FP\;+\;TN\;+\;FN\right)}\;\;\;\\ Recall\;or\;Sensitivity\;\left(\%\;of\;true\;positive\;identi\mathrm{fi}cation\right)=\frac{TP}{\left(TP\;+\;FN\right)} \end{array}$$

$$Specificity\;\left(\%\;of\;true\;negative\;\;identification\right)\;=\;\frac{TN}{\left(TN\;+\;FP\right)}$$

$$MCC\;\left(Measurement\;of\;prediction\;\;quality\right)\;=\;\frac{\left(TP\;\times \;TN-\;FP\;\times \;FN\;\right)}{\sqrt{\left(TP\;+\;FN\right)\left(TP\;+\;FP\right)\left(TN\;+\;FP\right)\left(TN\;+\;FN\right)}}$$

The criteria that maximized the above parameters were selected and evaluated on the test dataset. The selected optimal criteria were then used for filtering the predicted *P. multocida *proteins. Subsequently, consensus predictions from the three groups were integrated (Figure 1) and a single list of predicted OMPs generated.

### Manual confirmation of the predicted proteins

After the consensus prediction, each predicted protein was manually confirmed as being outer membrane-associated by using public database searches and sequence analyses (Figure 1). The PubMed database http://www.ncbi.nlm.nih.gov/pubmed was used to retrieve published experimental information relevant to the predicted proteins. The UniProt protein database http://www.uniprot.org/ was searched for homology and domain/motif, protein-protein interactions, and functional and structural predictions. Structural homology was examined by using the HHpred program http://toolkit.tuebingen.mpg.de/hhpred, [63]). The STRING protein interaction database http://string-db.org/ was used to identify whether the predicted proteins interacted with any known proteins or were members of any characterized pathways. Taken together, these analyses confirmed the proteins as confidently predicted putative OMP candidates (Figure 1).

### Analysis of filtered-out predicted proteins

The use of filtering criteria aims to reduce the number of false positive proteins. However, it may increase the probability of losing a small proportion of true positive proteins. Therefore, predicted proteins that were filtered out by the consensus criteria were integrated and also examined by manual confirmation to identify such lost true positive proteins (Figure 1).

### Physicochemical properties of the predicted OMPs

Physicochemical properties, e.g. molecular weight, length of protein sequence, theoretical pI, grand average of hydropathicity (GRAVY) score, aliphatic index, charge, number of β-strands and helices, of the putative OMPs were predicted by the ProtParam program http://expasy.org/tools/protparam.html, TMBETA-NET [13] and TMHMM [64].

## Availability and requirements

**Project name: **None

**Project home page: **None

**Operating system: **Platform independent

**Programming language: **Java, Perl

**Other requirements: **Excel

**License: **None for usage

**Any restrictions to use by non-academics: **None

## Competing interests

The authors declare that they have no competing interests.

## Authors' contributions

TE designed, developed and implemented the method. RD, RB and PH continuously supported the work and provided valuable comments. TE drafted the manuscript. All authors read, corrected and approved the final manuscript.

## Supplementary Material

Additional file 1 **Proteins predicted from the avian strain genome of *P. multocida *using 10 bioinformatic predictors**. 421 proteins were predicted using 10 bioinformatic predictors classified into three groups (four subcellular localization predictors, four transmembrane β-barrel protein predictors and two lipoprotein predictors) from the genome of avian *P. multocida *strain Pm70. "YES" indicates positive OMP prediction and "NO" indicates negative OMP prediction. The total number of positive predictions of each protein is summarized in the last column of each predictor group. The total number of positive predictions of each predictor is summarized in the last row. "-" represents unannotated genes. Click here for file

Additional file 2 **Proteins predicted from the porcine strain genome of *P. multocida *using 10 bioinformatic predictors**. 439 proteins were predicted using 10 bioinformatic predictors classified into three groups (four subcellular localization predictors, four transmembrane β-barrel protein predictors and two lipoprotein predictors) from the genome of porcine *P. multocida *strain 3480. "YES" indicates positive OMP prediction and "NO" indicates negative OMP prediction. The total number of positive predictions of each protein is summarized in the last column of each predictor group. The total number of positive predictions of each predictor is summarized in the last row. "-" represents unannotated genes. Click here for file

Additional file 3 **Training protein dataset used for two-fold cross-validation of consensus prediction**. Training dataset containing 526 Gram-negative bacterial protein sequences of known localization. Pairwise sequence comparison of these protein sequences showed less than 25% identity. This dataset was used for selection of various criteria of the consensus prediction using four statistical parameters including accuracy, recall/sensitivity, specificity and MCC. "OMP" indicates positive OMP prediction and "nonOMP" indicates non-OMP prediction. Total number of positive prediction of each protein is summarized in the last column of each predictor group. Total number of positive prediction of each predictor is summarized in the last row. "cyt" = cytoplasmic protein; "imp" = inner membrane protein; "per" = periplasmic protein; "omp" = outer membrane protein; and "ext" = extracellular protein. Click here for file

Additional file 4 **Test protein dataset used for two-fold cross-validation of consensus prediction**. Test dataset containing 529 Gram-negative bacterial protein sequences of known localization. Pairwise sequence comparison of these protein sequences showed less than 25% identity. This dataset was used for validation of selected criteria of the consensus prediction using four statistical parameters including accuracy, recall/sensitivity, specificity and MCC. "OMP" indicates positive OMP prediction and "nonOMP" indicates non-OMP prediction. Total number of positive predictions of each protein is summarized in the last column of each predictor group. Total number of positive predictions of each predictor is summarized in the last row. "cyt" = cytoplasmic protein; "imp" = inner membrane protein; "per" = periplasmic protein; "omp" = outer membrane protein; and "ext" = extracellular protein. Click here for file

Additional file 5 **Properties of confidently predicted OMPs**. Confidently-predicted putative OMPs identified from the genome of avian *P. multocida *strain Pm70 by 10 predictors, categorized into three groups (subcellular localization, transmembrane β-barrel and lipoprotein predictors) and subjected to the bioinformatic process described in Figure 1. OMPs predicted from the porcine *P. multocida *genome strain 3480 were compared. Physicochemical properties including molecular weight (MW), PI, aliphatic index, GRAVY score, number of transmembrane helices and β-strands, length and charge of each protein are also shown. Proteins were classified by predictor groups. Click here for file

Additional file 6 **Functional classification of the confidently predicted OMPs**. Functional classification of the 98 confidently predicted OMPs from the avian *P. multocida *genome. Click here for file
